# A review of recent advances on single use of antibody-drug conjugates or combination with tumor immunology therapy for gynecologic cancer

**DOI:** 10.3389/fphar.2022.1093666

**Published:** 2022-12-22

**Authors:** An-Jin Wang, Yang Gao, Yu-Ying Shi, Meng-Yuan Dai, Hong-Bing Cai

**Affiliations:** ^1^ Department of Gynecological Oncology, Zhongnan Hospital of Wuhan University, Wuhan, Hubei, China; ^2^ Hubei Key Laboratory of Tumor Biological Behaviors, Wuhan, Hubei, China; ^3^ Hubei Cancer Clinical Study Center, Wuhan, Hubei, China

**Keywords:** gynecologic oncology, immune checkpoint inhibitors (ICI), antibody-drug conjugates (ADC)s, mirvetuximab soravtansine (IMGN853), tisotumab vedotin

## Abstract

Immune checkpoint inhibitors have made significant progress in the treatment of various cancers. However, due to the low ICI responsive rate for the gynecologic cancer, ICI two-drug combination therapy tends to be a predominant way for clinical treatment. Antibody-drug conjugates, a promising therapeutic modality for cancer, have been approved by the FDA for breast cancer, lymphoma, multiple myeloma and gastric cancer. On September 2021, the FDA granted accelerated approval to tisotumab vedotin for patients with recurrent or metastatic cervical cancer. Currently, the role of therapy of ADCs on gynecologic tumors was also included in medication regimens. Now more than 30 ADCs targeting for 20 biomarkers are under clinical trials in the field, including monotherapy or combination with others for multiple lines of therapy. Some ADCs have been proved to enhance the antitumor immunity effect on both pre-clinical models and clinical trials. Therefore, combination of ADCs and ICIs are expected in clinical trials. In this review, we discuss current development of ADCs in gynecologic oncology and the combination effects of ICIs and ADCs.

## 1 Introduction

The first antibody-drug conjugate (ADC) drug, Mylotarg, was approved by the FDA in 2009. After a decade of advancement in conjugation biochemistry, a third-generation ADC drug, DS-8201, was approved in 2019, providing new possibilities for the use of ADC in cancer treatment. To date, ADCs have been approved for breast cancer, lymphoma, multiple myeloma, and gastric cancer and hundreds are in different phases of clinical trials for other tumor types (Beck et al., 2017; Abdollahpour-Alitappeh et al., 2019). In September 2021, tisotumab vedotin was granted FDA accelerated approval for recurrent or metastatic cervical cancer, becoming the 12th ADC approved by the FDA and the first for use against gynecologic cancers. Now more than 30 ADCs targeting 20 biomarkers are being tested in clinical trials, of which IMGN853 and tisotumab vedotin for ovarian and cervical cancer, respectively, are the first to be tested in phase III clinical trials. Over the last 10 years, cancer immunotherapy has undergone revolutionary changes. Immune checkpoint inhibitors (ICI), PD-1, PD-L1 and CTLA-4 monoclonal antibodies, represent major milestones in the treatment of human cancers, especially Hodgkin’s lymphoma and melanoma (Drakes et al., 2020; Kurnit et al., 2020). On October 2021, the FDA approved pembrolizumab (Keytruda) for use in combination with chemotherapy, with or without bevacizumab, for patients with persistent, recurrent, or metastatic cervical tumors that express PD-L1. On March 2022 pembrolizumab was FDA approved as a single agent for patients with advanced endometrial carcinoma that is microsatellite instability-high (MSI-H) or mismatch repair deficient (dMMR). However, the effect of ICIs on gynecologic cancer remain less than ideal. Combination ADC-ICI therapy is thought to be a potential solution.

## 2 ADC structure

ADCs contain biomarker-specific antibody, cytotoxic payloads and linkers (Ritchie et al., 2013). ADCs expand the therapeutic window by specifically delivering cytotoxic drugs to tumors and reducing their retention in healthy tissues (Tarcsa et al., 2020).

### 2.1 Antigen and antibody

Biomarkers are highly expressed in tumors and have little or no expression in normal tissues. IgG, in particular IgG1 and occasionally IgG2, IgG4, are often used in ADCs due to their long circulation half-life and high affinity. Some ADCs employ unique monoclonal antibodies such as STRO-002 with structures that are specifically designed for site-specific conjugation (Cheng et al., 2018).

### 2.2 Linkers

Linker stability *in vivo* ensures that cytotoxic molecules are not launched prematurely and that drug cleavage occurs once it enters the tumor, preventing systemic toxicity (Jain et al., 2015). There is a negative correlation between linker stability and ADC toxicity. The first marketed ADC, Mylotarg® (Gemtuzumab ozogamicin), was removed from the market in 2011 because its hydrazone linker was shown to be unstable causing high toxicity (van der Velden et al., 2001; Lu et al., 2016). Other recent cleavable linkers have increased stability, though off-target effects may persist. Cleavable linkers are more likely than non-cleavable linkers to cause bystander effects that contribute to tumor death (Chari et al., 2014; Burton et al., 2019).

The two types of linkers are shown in the Table 1. In addition, some ADCs such as DMUC4064A, XMT-1536, and stro-002, are being coupled by proprietary technologies (Ohri et al., 2018; Abrahams et al., 2019; Yurkovetskiy et al., 2021).

**TABLE 1:** Linkers types and features.

Cleavage	Linkers	Lability	Cleavage conditions	Features
Cleavable	Hydrazone bond	Acid-labile	Low pH in endosomes and lysosomes	Tend to cause bystander effects and off-target effects
	Peptide bond	Cathepsin B-labile	Cathepsin B, a lysosomal protein overexpressed in various cancer cells, for recognition and cleavage of specific peptide sequences	
	Disulfide bond	Glutathione-labile	Higher intracellular concentrations of glutathione than plasma	
Non-cleavable	Sulfide bond	Lysosome-labile	Completely degradation of linker and antibody in lysosomes	High cycling stability

### 2.3 Cytotoxic payloads

ADCs are dependent on the inclusion of highly cytotoxic molecules because the complex mechanisms by which ADCs function tends to reduce their utility. In addition, cytotoxic molecules have stable circulation and are easy to conjugate with antibodies (Teicher and Chari, 2011). The cytotoxic molecules used in ADCs are very limited, primarily falling into two categories, microtubule-targeting and DNA-damaging agents; however, the next-generation of ADCs is making use of RNA polymerase inhibitors and other agents (Pahl et al., 2018). Microtubule-targeting agents are the most widely used payloads, targeting maytansine sites (DM1 and DM4) and vinca alkaloid sites (MMAE and MMAF) to inhibit tubulin, disrupt microtubules, and arrest the cell cycle in the G2/M phase, inducing cell death (Chen et al., 2017). Importantly, these toxic properties are only functional in proliferating cells. In contrast, DNA-damaging agents are cytotoxic in both proliferating and non-proliferating cells since their mode of cytotoxic action is not dependent on the cell cycle. The categories of cytotoxic payloads are shown in the Table 2.

**TABLE 2:** Types of toxins used in ADCs.

ADC toxin types	Agents	Mechanism of action	Sensitive tumor cells
Microtubule inhibitors	MMAE, MMAF	Inhibit of microtubulin to blocks mitosis	Proliferating cancer cells
	DM1, DM4		
	Novel microtubule inhibitors		
DNA damaging agent	Psilocybin	Intercalate DNA to inhibits topoisomerase I	Rapidly proliferating and relatively non-proliferating
	Gliclazomycin, streptomycin	Bind to DNA minor grooves to induces DNA double-strand breakage	
	PBD, doxorubicin	DNA alkylation	
	Adriamycin	Topoisomerase II inhibitor	
RNA polymerase inhibitors	α-amanitin	Block DNA transcription	——
Other	Bcl-xL inhibitors, RNA spliceosome inhibitors, TLR 7/8 dual agonistetc.	——	——

## 3 Use of ADCs in gynecologic cancer

**FIGURE 1:**
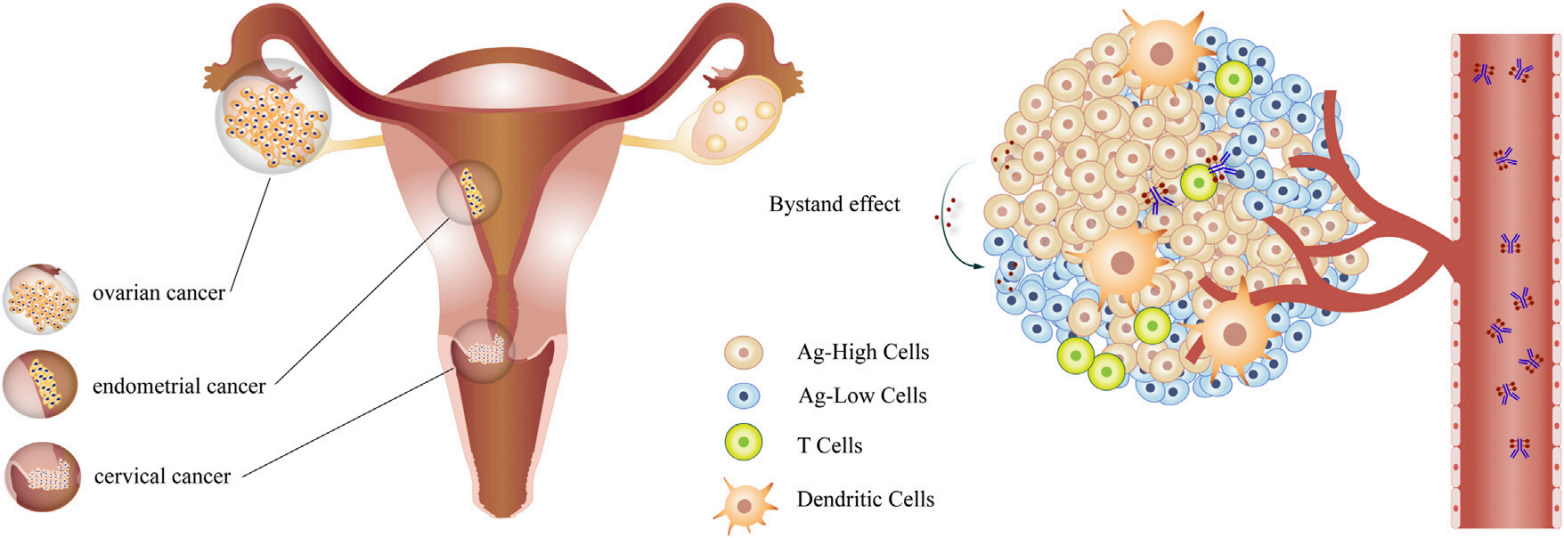
The ADCs in gynecologic cancer ADCs target antigen-high cells in gynecological tumors through the circulation and kill antigen-low cells by bystander effects.

The function of ADC drugs against refractory gynecologic cancers is receiving increasing attention. Clinical trials for some biomarkers are in progress. The Table 3 summarizes the ADCs of gynecological oncology in clinical trials (Figure 1).

**TABLE 3:** ADCs in gynecologic oncology in clinical trial.

Targets	ADC	Antibodies	Linkers	Payloads	Drug-antibody ratio	Clinical trial phase	Gynecologic cancer mainly face to	Ocular toxicity
FRα	Mirvetuximab soravtansine	Humanized IgG1	Sulfo-SPDB (disulfide linker)	DM4	3.5	III, granted priority review	Ovarian cancer and endometrial	Observed
	MORAb-202	Farletuzumab	Cathepsin-B cleavable linker	Eribulin	4.1	I/II	Ovarian cancer and endometrial	Observed, infrequently
	STRO-002	Humanized IgG1	Protease-labile Val-Cit-PABA	Hemiasterlin	4	I	Ovarian cancer	Unobserved
Mesothelin	Anetumab ravtansine	Humanized IgG1	Disulfide linker	DM4	3.2	II	Ovarian cancer	Observed
	DMOT4039A	Humanized IgG1	Protease-labile valine-citrulline linker	MMAE	3.5	I	Ovarian cancer	Unobserved
	BMS-986148	Humanized IgG1	Protease-labile valine-citrulline linker	Tubulysin	3	I/II	Ovarian cancer	Observed
	RC88	Undisclosed	C75:Py-MAA-Val-Cit-PAB	MMAE	/	I/IIa	Ovarian cancer	Undisclosed
MUC16	DMUC5754A	Humanized IgG1	Protease-labile MAA-Val-Cit-PAB linker	MMAE	3.5	I	Ovarian cancer	Unobserved
NaPi2B	XMT1536	Humanized IgG1	Protease-labile linker	Auristatin F- hydroxypropylamide (AF-HPA)	10–15	I/II	ovarian cancer and endometrial cancer	Unobserved
	Lifastuzumab vedotin	Humanized IgG1	Protease-labile Val-Cit-PABA	MMAE	4	II, did not meet primary endpoint	Ovarian cancer and endometrial cancer	Observed, infrequently
Tissue factor	Tisotumab vedotin	Humanized IgG1	Protease-labile mc-val-cit-PABC linker	MMAE	4	III, accelerate approval	Cervical cancer	Observed
PTK7	PF-06647020	Humanized Immunoglobulin	Protease-labile valine-citrulline linker	Auristatin-0101	4	I/II	Ovarian cancer	Unobserved
Trop2	Sacituzumab govitecan	Humanized IgG	Acid-labile linker, CLA2 linker	SN-38	7.6	I/II basket trial (FDA approved for breast cancer)	Endometrial cancer, EOC and cervical cancer	Unobserved
HER2	Ado-tratuzumab emtansine	Trastuzumab	Non-cleavable thioether linker	DM1	3.5	I/II(FDA approved for breast cancer)	Endometrial cancer and ovarian cancer	Unobserved
	BDC-1001	Trastuzumab	Non-cleavable thioether linker	TLR 7/8 dual agonist	/	I/II	Endometrial cancer and ovarian cancer	Unobserved
	Trastuzumab Deruxtecan	Trastuzumab	Lysosomal cathepsins-cleavable tetrapeptide linker	Deruxtecan	8	I/II(FDA approved for breast cancer)	Ovarian, endometrial, and cervical cancer	Unobserved
	Trastuzumab duocarmycin	Trastuzumab	Lysosomally cleavable linker	Duocarmycin	2.8	I/II(FDA approved for breast cancer)	Endometrial cancer	Observed
	A166	Trastuzumab	Protease-labile valine-citrulline linker	MMAF derivative (Duostatin-5)	2	I/II	Cervical cancer	Observed
ALCAM/CD116	CX2009	Probody	Sulfo-SPDB(disulfide linker)	DM4	3.5	I/II	Ovarian and endometrial cancer	Observed
CEACAM5	SAR408701 (Tusamitamab ravtansine)	Humanized IgG1	Sulfo-SPDB(disulfide linker)	DM4	3.8	I/II	Ovarian cancer	Observed

### 3.1 FRα

Alpha folate receptor binding antibody (FRα) is a 38–40 kDa glycosyl-phosphatidylinositol (GPI) glycoprotein anchored to the cell surface that binds folate for intracellular transport (Elnakat and Ratnam, 2004; Kitamura et al., 2010). Normally, the expression of FRα is restricted to a small number of polarized epithelia in the choroid plexus, lung, kidney, uterus, and placenta, while it is overexpressed in the majority of ovarian, uterine, and ependymal brain tumors and in varying percentages of lung, breast, kidney and colon carcinomas (Elnakat and Ratnam, 2006; Birrer et al., 2019; Salazar and Ratnam, 2007; Scaranti et al., 2020; O'Shannessy et al., 2012). FRα is expressed in >80% of epithelial ovarian cancers (EOC) and is associated with poor prognosis of EOC (Ross et al., 1994; Kelemen, 2006; Vergote et al., 2015).

#### 3.1.1 Mirvetuximab soravtansine

Mirvetuximab soravtansine (IMGN853), an advanced ADC in gynecological oncology, includes an FRα binding antibody, a cleavable disulfide link, and a maytansinoid DM4 payload (Ab et al., 2015). A phase I dose-escalation trial of IMGN853 has a favorable safety profile. The most common adverse events (AEs) were fatigue (25%), blurred vision (23%), diarrhea (21%), and peripheral neuropathy (21%), the majority of which were grades 1 or 2. Treatment-related serious adverse events (SAE) (9%) included grade 3 hypophosphatemia, punctate keratitis, episode, corneal opacity, and pulmonary edema. The blurred vision and keratopathy were reversible. All 44 patients who received IMGN853 were not required with expression of FRα. Of 43 assessable patients, two EOC patients had confirmed partial responses (PRs) with an the objective response rate (ORR) of 5%, 22 patients had stable disease (SD) and five patients (four with EOC and one with endometrial cancer) had a confirmed CA 125 response with an overall clinical benefit rate (ORR + SD ≥4 months + CA 125 response) of 23% (Moore et al., 2017a). The phase I expansion cohort study included 46 patients for whom ≥ 25% of their tumor cells had at least 2+ FRα staining intensity. The ORR was 26%, including one complete response (CR) and 11 PRs, the median progression-free survival (PFS) was 4.8 months, and the median duration of response (DoR) was 19.1 weeks. Remarkably, there appeared to be a relationship between the receipt of prior lines of therapy and the response to IMGN853. An ORR of 39% was observed in patients who had received one to three prior lines, compared with 13% among patients who had received at least four (Moore et al., 2017b). The overall ORR in a phase Ib study was 22%, including two CRs and four PRs. This study also revealed a correlation between FRα expression and clinical efficacy. Low-expression cases had no objective response, with a median PFS of 2.8 months, while the ORR of the medium-expression cohort was 20% (1/5), with a median PFS of 3.9 months and the ORR of the high-expression cohort was 31% (5/16, including two CRs) with a median PFS of 5.4 months (Martin et al., 2017).

These early trials provided sufficient evidence to inform the inclusion criteria of FORWARD I (NCT02631876), a phase III monotherapy trial for patients with platinum-resistant FRα positive advanced EOC, primary peritoneal cancer, and/or fallopian tube cancer. A total of 336 patients were randomized to receive IMGN853 or chemotherapy (paclitaxel, pegylated liposomal doxorubicin, or topotecan) in a 2:1 ratio. The IMGN853 and chemotherapy groups had ORRs of 22% and 12%, respectively; however, both the PFS (HR = 0.98, *p* = 0.897), the primary endpoint, and the overall survival (HR = 0.81, *p* = 0.248) were not significantly different. While not reaching the primary endpoint, IMGN853 demonstrated superior clinical activity and fewer adverse events than chemotherapy (Moore et al., 2021). Two new Phase III studies, SORAYA (NCT04296890) and MIRASOL (NCT04209855) based on FORWARD I, are in progress (Moore et al., 2021).

#### 3.1.2 MORAb-202

MORAb-202 is an ADC in which farletuzumab is bonded to eribulin by a cathepsin-B cleavable linker (Cheng et al., 2018). As a human monoclonal antibody specific for FRα, farletuzumab combined with standard chemotherapy did not meet the primary endpoint in a phase III trial for platinum-sensitive recurrent ovarian cancer (Vergote et al., 2016). This phase I study included 22 patients with FRα-positive solid tumors, including 12 patients with ovarian cancer. A total of 21 of 22 patients (95%) experienced treatment-emergent AEs, ten (45%) of whom developed leukopenia and neutropenia, the most frequent adverse events. One patient experienced a grade 3 rise in alanine aminotransferase and γ-glutamyl transferase as dose-limiting toxicities and five patients (23%) developed pneumonitis/interstitial lung disease related to MORAb-202. The overall ORR of the study was 45.45% (10/22) including one CR, nine PRs, and eight (36%) SDs. Notably, normalized serum FRα was associated with the highest level of tumor shrinkage (Shimizu et al., 2021). Phase I/II studies (NCT04300556) to further assess MORAb-202 are ongoing.

#### 3.1.3 STRO-002

STRO-002 is a novel FRα-targeting ADC that includes the tubulin-targeting 3-aminophenyl hemiasterlin warhead, SC209, joined to the antibody, SP8166. This drug is generated using a cell-free antibody production system (XpressCF™) and a site-specific conjugation (XpressCF+™) platform (Abrahams et al., 2019). A phase I dose-escalation study of STRO-002 (NCT03748186) contained 39 platinum-resistant patients without requirement for FRα expression. Most (86%) treatment-emergent AEs were grades 1 or 2. The most common treatment-related grade 3 and 4 AEs were reversible neutrophil reduction (36%), neutropenia (33%), arthralgia (12.8%), and neuropathy (7.7%). Significantly, no ocular toxicity signals have been observed, distinguishing STRO-002 from other ADCs. Most ADCs revealed ocular toxicities that are driven by the payload present and not by antigen expression. And the most commonly reported cases above are ADCs that contain DM4 or MMAF whose biomarkers barely express in the eye (Eaton et al., 2015). Within assessable patients, an ORR of 32% (10/31) was observed, including one CR (3%), four confirmed PRs, and five unconfirmed PRs, with a median PFS of 7.2 months and a median DoR of 5.8 weeks. There were also 18 SDs (58%) and 3 PDs (9.67%) (Naumann et al., 2021).

### 3.2 Mesothelin

Mesothelin is a 40-kDa glycoprotein that is attached to the cell membrane by a glycosylphosphatidylinositol (GPI) (Hassan et al., 2004; Pastan and Hassan, 2014). Under physiological conditions, mesothelin is expressed in the upper part of the vagina, the uterus, and the fallopian tubes developed from the mesodermal Müllerian (paramesonephric) duct. Expression of mesothelin increases substantially during malignant transformation (Chang and Pastan, 1996; Jirsova et al., 2010), in particular, that associated with pancreatic cancer, mesothelioma, lung cancer, pancreatic cancer, breast cancer, ovarian cancer, endometrial cancer and cervical adenocarcinoma (Argani et al., 2001; Tchou et al., 2012; Iizuka et al., 2013; Qiao et al., 2014; Qiao et al., 2016; Stewart and Cristea, 2019; Weidemann et al., 2021). Mesothelin expression increases in 70%–85% ovarian tumors, making it a potential target for this disease (Hassan et al., 2005; Stewart and Cristea, 2019).

#### 3.2.1 Anetumab ravtansine (BAY 94-9343)

Anetumab Ravtansine is an ADC consisting of maytansinoid DM-4 conjugated to an anti-mesothelin-monoclonal antibody IgG1 *via* a disulfide-containing linker [a reducible SPDB linker (N-succinimidyl 4-(2-pyridyldithio) butanoate)]. A total of 148 patients with high-expression mesothelin cancer, including 21 with ovarian cancer, participated in phase I clinical trials (NCT01439152) for anetumab ravtansine. At the q3w maximum tolerated dose (MTD) (6.5 mg/kg), the most common drug-related AEs were fatigue, nausea, diarrhea, anorexia, vomiting, and peripheral sensory neuropathy, and the PR, SD, and DCR for ovarian cancer patients were 5%, 52%, and 57%, respectively, with a median DoR of 62 days and the median PFS of 2.8 months (Hassan et al., 2020). In a subsequent phase Ib study, anetumab ravtansine was combined with pegylated liposomal doxorubicin in platinum-resistant ovarian, fallopian tube, or primary peritoneal cancer. Within 21 assessable patients, the disease control rate (DCR) was 86% of 11 PRs (52%) and 7 SDs (33%), and a durable PR (>250 days) was observed in six patients (29%) (Santin et al., 2020).

#### 3.2.2 DMOT4039A

DMOT4039A is composed of the humanized IgG1 anti-mesothelin mAb, h7D9.v3, and monomethyl auristatin E (MMAE) combined with a protease-labile valine-citrulline linker (Scales et al., 2014). In a phase I study of patients with unresectable pancreatic or platinum-resistant ovarian cancer, 31 ovarian cancer patients had strong staining for mesothelin. The main AEs were gastrointestinal or constitutional. Cumulative peripheral neuropathy (grades 1–3) resulting from microtubule inhibitor-specific toxicity, occurred in 20% of patients. At the q3w RP2D, three of ten ovarian cancer patients revealed confirmed partial responses, with durations of 2.7, 3.6, and 4.1 months and an additional three patients showed a CA125 response without a RECIST response. The median PFS for ovarian cancer patients at RP2D was 4.9 months. The trial also found that both serum mesothelin levels and tissue mesothelin immunohistochemistry (IHC) scores were not associated with the clinical activity of DMOT4039A (Weekes et al., 2016).

#### 3.2.3 BMS-986148

BMS-986148 contains tubulysin, a cytotoxic peptide with antimitotic activity that is attached to a fully human IgG1 anti-mesothelin mAb *via* a valine-citrulline linker (Clarke et al., 2019). A phase I/IIa trial (CA008-002, NCT02341625) assessed the safety, tolerability, and preliminary efficacy of BMS-986148 ± nivolumab against mesothelioma, ovarian cancer, pancreatic cancer, gastric cancer, and non-small cell lung cancer (NSCLC). Hepatic TRAEs occurred in all treatment cohorts, the most common (≥10%) of which were elevated aspartate aminotransferase, alanine aminotransferase, and alkaline phosphatase. On study day 130, an ovarian cancer patient died from pneumonitis related BMS-986148 monotherapy at the MTD level. The ORR and DCR were 9% and 13%, respectively, including two PRs (DOR, 19.91 and 3.02 months) and 11 SDs among 22 ovarian cancer patients, with a PFS of 2.8 months. There were also seven PDs (Rottey et al., 2022).

#### 3.2.4 RC88

RC88, composed of anti-mesothelin mAb and MMAE, is being tested in a phase I clinical trial in mesothelin-positive solid tumor patients, including those with ovarian cancer (Jiang et al., 2021).

### 3.3 MUC16 (CA125)

MUC16, a large type I transmembrane mucin of the MUC family (Yin and Lloyd, 2001; Thériault et al., 2011; Aithal et al., 2018), is a precursor of the most widely used biomarker for recurrent ovarian cancer, CA125. MUC16 is overexpressed in the majority (80%) of human EOCs but not in the epithelium of normal ovaries, and also plays a role in endometrial, fallopian tube, pancreatic, colon, peritoneal, nasopharyngeal, lung, breast and stomach cancers (Bast et al., 1981; Kabawat et al., 1983; Macdonald et al., 1988; Rosen et al., 2005).

#### 3.3.1 DMUC5754A (sofituzumab vedotin, RG7458)

DMUC5754A contains the humanized IgG1 anti-MUC16 monoclonal antibody and MMAE linked through a protease-labile linker, maleimidocaproyl-valine-citrulline-p-aminobenzyloxycarbonyl (Chen et al., 2007). A phase I dose-escalation trial of DMUC5754A in 66 ovarian cancer and 11 pancreatic cancer patients found that fatigue, peripheral neuropathy, nausea, decreased appetite, vomiting, diarrhea, alopecia, pyrexia, anemia, neutropenia, alopecia, decreased appetite and hypomagnesemia were the most common AEs across all drug dose levels. Drug-related SAEs included small intestine obstruction, hypocalcemia, neutropenia, dehydration, diarrhea, nausea, and posterior reversible encephalopathy syndrome (PRES). The only case of PRES was reversed following drug cessation. No ocular toxicity signals were reported. The ORR was 11%, including one CR and six PRs, and there were six additional SDs lasting >6 months. Notably, the objective responses were only observed in MUC16-high patients (Liu et al., 2016).

#### 3.3.2 DMUC4064A

DMUC4064A contains a humanized IgG1 anti-MUC16 monoclonal antibody (MMUC3333A) and two MMAE connected by a protease-cleavable linker. Using the new technology, “THIOMAB™ drug conjugates” (TDC) for site-directed conjugation, DMUC4064A was shown to have a more homogenous payload than other ADCs (Ohri et al., 2018). A phase I dose-escalation trial of DMUC4064A in patients with platinum-resistant ovarian cancer found that fatigue, nausea, abdominal pain, constipation, blurred vision, diarrhea, and anemia were the most common AEs. Ocular AE related to DMUC4064A, including blurred vision, dry eye, keratitis, cataract, corneal epithelial microcysts, eye pain, and photophobia, occurred in 40% of patients. A total of 27 SAEs occurred in 25% of patients including one death due to septic shock. The trial was associated with one CR and 20 PRs. An ORR of 46% was observed in the 54 patients with a high MUC16 IHC score of 2 + or 3+ and, an overall median PFS of 3.9 months. There were also 19 SDs (35%) and 12 PDs (22%). The best responses were SD (*n* = 4, 57%) and PD (*n* = 3, 43%) in seven patients with MUC16 IHC scores of 0 or 1+ (Liu et al., 2021). However, clinical development of DMUC4064A has been discontinued for non-safety-related reasons.

### 3.4 NaPi2b

NaPi2b is a multitransmembrane protein involved in transcellular inorganic phosphate absorption and the maintenance of phosphate homeostasis (Xu et al., 1999). NaPi2b is also associated with cell differentiation and tumorigenesis and is broadly expressed in human lung, ovarian, and thyroid cancers (Andersson et al., 2009; Finstad et al., 1997; Lin et al., 2015; Rangel et al., 2003; Jarząb et al., 2005; Suzuki et al., 2011; Xiao, 2005). Differential expression between tumors and normal tissues, cell surface localization and endocytosis make NaPi2b a potential target for ADC design.

#### 3.4.1 XMT-1536

XMT-1536 (upifitamab rilsodotin) is a first-in-class Dolaflexin ADC, employing the Dolaflexin platform to provide ten DolaLock auristatin payload molecules per anti-NaPi2b antibody (Yurkovetskiy et al., 2021). In a phase I study (NCT03319628), fatigue, nausea, vomiting, pyrexia, decreased appetite, diarrhea, and a transient increase in AST were the most common treatment-related AEs. The ORR was 39%, including two CRs, and the DCR was 81% in 26 ovarian cancer patients with high NaPi2b expression. The two CR patients had previously received bevacizumab and PARPi treatment. More than 60% of patients had high expression of NaPi2b (Hamilton et al., 2020; Richardson et al., 2020; Concin et al., 2021; Richardson et al., 2021).

#### 3.4.2 Lifastuzumab vedotin (DNIB0600A)

Lifastuzumab Vedotin is an ADC that includes a humanized IgG1 anti-NaPi2b monoclonal antibody (MNIB2126A) and a MMAE that are connected by a protease-labile linker, maleimidocaproyl-valine-citrulline-p-aminobenzyloxycarbonyl. A phase Ia study (NCT01363947) of lifastuzumab vedotin in NSCLC or platinum-resistant ovarian cancer patients assessed its safety and preliminary antitumor activity. Most (89%) patients experienced AEs related to the study drug, the most common of which were fatigue (52%), nausea (38%), decreased appetite (33%), peripheral sensory neuropathy (29%), and vomiting (24%). A total of 16% of patients who received the 2.4 mg/kg dose stopped treatment due to AEs and one dose-limiting toxicity (DLT) occurred at 1.8 mg/kg. This study established the RP2D at 2.4 mg/kg, identical to the RP2D of most Genentech MMAE-ADCs with similar drug-antibody ratios. Peripheral neuropathy associated with MMAE was reported in 41% of patients and 54 patients (63%) experienced grade 1–3 pulmonary toxicity, a common risk attributed to ADCs. At active doses of ≥1.8 mg/kg, partial responses were observed in 11 of 24 (46%) patients with PROC, all of which were NaPi2b-high, with DoR ranging from 43 to 561 days (median, 342.0) (Gerber et al., 2020). In a randomized phase II study (NCT01991210), lifastuzumab vedotin (DNIB0600A) was compared with pegylated liposomal doxorubicin in patients with platinum-resistant ovarian cancer. While a higher ORR was observed in patients treated with Lifastuzumab than liposomal doxorubicin (34% *versus* 15%, respectively), the study did not reach its end point because the median PFS (5.3 *versus* 3.1 months, respectively) was not statistically significant (Banerjee et al., 2018). As a result, Lifastuzumab Vedotin has not been studied further.

### 3.5 Tissue factor

Tissue factor (TF), also known as thromboplastine, is the main physiological initiator of the extrinsic coagulation pathway (Bogdanov et al., 2003; Yu and Rak, 2004). In adults, TF is constitutively expressed in cells of the subendothelial vessel wall, including smooth muscle cells, pericytes, and fibroblasts (Förster et al., 2006; Pan et al., 2019). However, most cancer patients show hypercoagulability, and TF is involved in cancer cell proliferation, survival, angiogenesis, and the epithelial-to-mesenchymal that promotes tumor development (Magnus et al., 2014; Alley et al., 2019). TF is more highly expressed in the malignant tissue of the ovaries, cervix uteri, and uterus than in healthy tissue and is also expressed in solid cancers of the pancreas, lung, prostate, bladder, breast, and colon (Cocco et al., 2010; Cocco et al., 2011; Zhao et al., 2018).

#### 3.5.1 Tisotumab vedotin (HuMax-TF-ADC, or TF-011-MMAE)

Tisotumab vedotin (TV) is an ADC that was granted accelerated approval in the US for the treatment of recurrent or metastatic cervical cancer with disease progression on or after chemotherapy. Tisotumab vedotin is comprised of an anti-TF-monoclonal antibody IgG1 and MMAE that are combined with a protease-cleavable linker (Alley et al., 2019). The first-in-human, multicenter, phase I–II (InnovaTV 201, NCT02001623) trial of tisotumab vedotin in patients with advanced or metastatic solid tumors that included relapsed, advanced, or metastatic cancers of the ovary, cervix, endometrium, bladder, prostate, or oesophagus, and squamous cell carcinoma of the head and neck or NSCLC. A total of 39 (27%) of 147 patients experienced a treatment-emergent serious AE associated with the study drug and one patient with pneumonia was considered possibly treatment-related. AEs of interest included bleeding, neuropathy, and ocular events (conjunctivitis, ulceration, keratitis, and symblepharon), which are characteristic of ADCs. Grade 1 epistaxis was most common, occurring in 98% of patients. Many (63/147; 43%) patients developed neuropathy, 51 (81%) of whom had received prior taxane chemotherapy. A total of 88 (60%) patients had an ocular event and 63 patients experienced conjunctivitis, some of which was relieved using ocular mitigation strategies. The ORR was 26.5% (9/34) for cervical cancer, 7.1% (1/14) for endometrial cancer, and 13.9% (5/36) for ovarian cancer (de Bono et al., 2019).

InnovaTV 204 (NCT03438396), a phase II study of tisotumab vedotin in patients with previously treated recurrent or metastatic cervical cancer revealed serious treatment-related AEs in 13 (13%) patients, including one death due to septic shock. The stable disease rate was 49% and the ORR was 24%, including seven (7%) CRs and 17 (17%) PRs. The DOR was 8.3 months, the median PFS was 4.2 months and the median OS was 12.1 months, with a 6-month PFS rate of 30% and a 6-month overall survival rate of 79% (Coleman et al., 2021). A randomized, phase III open-label study of this drug for cervical cancer patients and clinical studies of its use against other solid tumors are ongoing.

### 3.6 PTK7

Protein tyrosine kinase 7 (PTK7) is a highly conserved transmembrane PTK involved in Wnt signaling during haematopoietic and somatic progenitor cell development (Puppo et al., 2011; Peradziryi et al., 2012; Hayes et al., 2013; Hashmi et al., 2016; Huang et al., 2020). PTK7 is overexpressed in multiple tumor types, including advanced NSCLC, ovarian cancer, triple-negative breast cancer (TNBC), colon, gastric, and esophageal cancers (Shin et al., 2013; Chen et al., 2014; Gärtner et al., 2014; Haiyan et al., 2014; Lhoumeau et al., 2015; Jiang et al., 2019). PTK7 has no catalytic activity so cannot be used as an inhibitor but could serve as a target for ADCs (Damelin et al., 2017).

#### 3.6.1 PF-06647020 (Cofetuzumab Pelidotin)

PF-06647020 (Cofetuzumab Pelidotin) is an ADC comprised of a humanized anti-PTK7 mAb joined to the microtubule targeting drug, auristatin-0101 (Aur0101), by a cleavable valine-citrulline based linker (Sachdev et al., 2018). The first phase I trial (NCT02222922) involved 63 women with platinum-resistant ovarian cancer. The most common TRAEs were nausea, alopecia, fatigue, headache, neutropenia, and vomiting, 8% of patients developed grade 3 peripheral sensory neuropathy across dose levels, and two patients experienced grade 3 abdominal pain (DLT) at the highest dose. Grade 2–3 abdominal pain of unknown etiology has also been previously reported in the trial of the auristatin-based ADC, PF-06650808 (Rosen et al., 2020). The ORR was 27% in ovarian cancer patients (*n* = 63), 19% in NSCLC patients (*n* = 31), and 21% in TNBC patients (*n* = 29). The clinical effect of PF-06647020 correlated with PTK7 expression in tumor tissues (Maitland et al., 2021).

### 3.7 Trop2

Trop2, a tumor-associated calcium signal transducer of a human trophoblast cell, is overexpressed in a variety of human epithelial cancers (Basu et al., 1995). Trop2 ADCs have been primarily researched for use against breast cancer. However, overexpression has also been observed in patients with ovarian, endometrial, and cervical cancers (Bignotti et al., 2010; Varughese et al., 2011a; Varughese et al., 2011b; Raji et al., 2011). Preliminary findings suggest that Trop2 may be a promising target in gynecological oncology (Syed, 2020).

#### 3.7.1 Sacituzumab govitecan (IMMU-132)

Sacituzumab govitecan was approved by the FDA in 2021 as an ADC for mTNBC patients. This drug consists of a humanized anti-Trop2 monoclonal antibody and an SN-38 combined with a cleavable linker (Cardillo et al., 2015; Goldenberg et al., 2015). In a phase I/II basket trial (NCT01631552) of Sacituzumab govitecan, 483 pts (97.6%) experienced a treatment-related AE. The most common TRAEs included nausea (62.6%), diarrhea (56.2%), fatigue (48.3%), alopecia (40.4%), and neutropenia (57.8%), while the most prevalent treatment-related SAEs included febrile neutropenia (4.0%), diarrhea (2.8%), vomiting (1.4%), neutropenia (1.4%), and nausea (1.2%). Interestingly, treatment-related grade >2 neuropathy, serious cardiotoxicity, and ocular toxicity have not been reported. In this trial, 18 patients with platinum-resistant endometrial cancer responded more favorably to Sacituzumab govitecan than single-agent chemotherapy. The ORR of the endometrial cancer cohort was 22.2%, including four PRs. There are six SDs with a median OS of 11.9 months and a PFS of 3.2 months. The ORR was zero among eight EOC and one cervical cancer patients (Bardia et al., 2021).

#### 3.7.2 PF-06664178 (RN927C)

PF-06664178 is an ADC that combines a humanized anti-Trop2 IgG1 antibody (PF-06478924, RN926) with an AcLys-VCAur0101 (PF-06380101), a potent inhibitor of tubulin polymerization, *via* an enzymatic process (Strop et al., 2013; Strop et al., 2016). A phase I dose-escalation study of PF-06664178 involved 31 patients with solid tumors, including six with ovarian and one with cervical cancer. However, skin and mucosal cells were shown to have a particular sensitivity to the Aur0101 payload, causing intolerable skin rashes and inflammation of the mucous membranes along with neutropenia at a dose associated with minimal anti-tumor activity. As a result, the development of this drug has been discontinued (King et al., 2018).

### 3.8 HER2

HER2, a transmembrane protein with tyrosine kinase activity, is overexpressed in many tumor types, especially breast cancer, where expression is linked to poor prognosis, resistance to chemotherapy, hormone therapy, and radiotherapy, and an elevated risk of metastasis and recurrence (Bookman et al., 2003; Liu et al., 2015; Zhou et al., 2015; Connell and Doherty, 2017; Yang et al., 2021). Recent whole-exome sequencing and confirmatory IHC studies show high HER2/neu expression in ∼35% of patients with uterine serous carcinoma (USC) (Zhao et al., 2013; Rottmann et al., 2020).

#### 3.8.1 Ado-trastuzumab emtansine (T-DM1)

Ado-trastuzumab emtansine is FDA approved for use against HER2-positive breast cancer. This agent is composed of trastuzumab and 3–4 DM1 combined by a non-cleavable thioether linker (Verma et al., 2012). A phase II clinical trial (NCT02465060), NCI-MATCH (EAY131), involved 38 patients who had tumors with an ERRB2 copy number >7. Of the 36 evaluable patients, 8/10 (80%) patients with ovarian and endometrial cancer had stable disease with a median disease duration of 4.6 months. NGS measurements associated increased levels of the CN gene with a greater antitumor effect (Jhaveri et al., 2019). In an additional multi-histology basket trial (NCT02675829) of 58 patients with advanced lung, endometrial, salivary gland, biliary tract, ovarian, bladder, colorectal and other cancers, the overall ORR was 26% (14/53), with a lung cancer ORR of 50% (3/6), an endometrial cancer ORR of 22% (4/18, 2 CR), an ovarian cancer ORR of 17% (1/6), a salivary cancer ORR of 100% (5/5, 3 CR) and a biliary tract cancer ORR of 17% (1/6). There are also two clinical trials of T-DM1 in multi-drug combination for recurrent ovarian cancer and recurrent endometrial cancer (Li et al., 2018).

#### 3.8.2 BDC-1001

BDC-1001 is an immune-stimulating antibody conjugate (ISAC) drug that targets HER2. This drug contains an anti-HER2 IgG1 (trastuzumab bioanalogue) as the targeting component and the toxin present in conventional ADCs has been replaced with a toll-like receptor (TLR) 7/8 dual agonist that is coupled by a non-cleavable linker. TLR7/8 is important in innate immunity, helping to bridge non-specific and specific immune responses. TLR7/8 agonists attached to BDC-1001 are phagocytosed into lysosomes on HER2-positive tumor cells, activating myeloid APCs and presenting tumor-associated antigens to T cells (Ackerman et al., 2019). Phase 1/2 clinical data has revealed that fatigue, infusion-related reactions, nausea, abdominal pain, fever, arthralgia, constipation, anemia, diarrhea, dyspnea, and vomiting are the most common AEs associated with BDC-1001 (NCT04278144). DLTs have not been observed, and the q3w MTD was not achieved at 20 mg/kg. Many patients (42.1%) experienced AEs greater than grade 3, and 19 (33.3%) patients experienced SAEs, two of which were considered treatment-related. Of the 40 evaluable patients, the overall ORR was only 2.5% (one PR), with a disease control rate (DCR) of 32.5%. Twelve patients had SDs, including one with endometrial cancer for 24 months, one with cervical cancer for 23 + months, and one with ovarian cancer for 6 months (Dumbrava et al., 2021; Sharma et al., 2021).

#### 3.8.3 Trastuzumab deruxtecan (DS-8201, T-DXd)

DS-8201 consists of trastuzumab and an exatecan derivative topoisomerase I inhibitor, combined by a cleavable tetrapeptide linker that is linked to the antibody *via* a cysteine residue. This drug was approved by the FDA in 2019 to treat HER2-positive breast cancer. The phase II clinical trial, STATICE (UMIN00002956, NCCH1615), included 34 patients with HER2-positive uterine cancer sarcoma, of whom 22 had 2 + or 3 + HER2 expression and ten had 1 + expression. Of those with 2 + or 3 + expression, the objective remission rate was 55% (12/22), ten had stable disease, none had progression, and the disease control rate was 100%. Of those with 1 + HER2 expression, the ORR was 70% (7/10), three had stable disease, none had progression, and the DCR was 100% (Hasegawa et al., 2021). Another multicenter, multicohort, phase II study (NCT04482309) is ongoing and includes seven groups of patients (∼40 patients/group) with urothelial bladder, biliary tract, cervical, endometrial, ovarian, pancreatic, or rare tumors (Meric-Bernstam et al., 2022).

#### 3.8.4 Trastuzumab duocarmazine (SYD985)

Trastuzumab duocarmazine consists of Trastuzumab and duocarmazine connected by a cleavable linker (Black et al., 2016). A phase I/II trial (NCT02277717) assessed the use of Trastuzumab duocarmazine against multiple HER2-positive advanced solid tumors. Treatment-related SAEs were reported in 11% of patients during the dose-expansion phase, the most frequent of which were infusion-related reactions and dyspnea. The most common AEs were fatigue, conjunctivitis, and dry eye. Most patients (104/146; 71%) had at least one ocular AE, and one dose-limiting pneumonia death was associated with the highest treatment dose. In the non-breast cancer extension cohort, 25 of 45 evaluable patients (57%) had a reduction in target lesions. The ORR was 6% and 25% among patients with gastric cancer and uroepithelial cancer, respectively. A total of 5 of 13 patients (39%) with endometrial cancer had an ORR and a median PFS of 4–3 months (2.4–9.9) (Banerji et al., 2019). A phase II trial of SYD985 for the treatment of HER2-positive endometrial cancer (NCT04205630) and a phase I trial of this drug in combination with niraparib (NCT04235101) for the treatment of HER2-positive solid tumors are currently ongoing.

#### 3.8.5 A166

A166 is a monoclonal antibody specific for HER2 that is coupled with distortion derived from MMAF-5. A phase I–II clinical study (NCT03602079) is underway among patients with HER2-positive solid tumors, including those with cervical cancer. Preliminary findings suggest that the drug is well tolerated; however, eye toxicity, including dry eye syndrome and blurred vision, was common. SD and PR were observed in almost 60% of patients (Liu et al., 2020).

**FIGURE 2:**
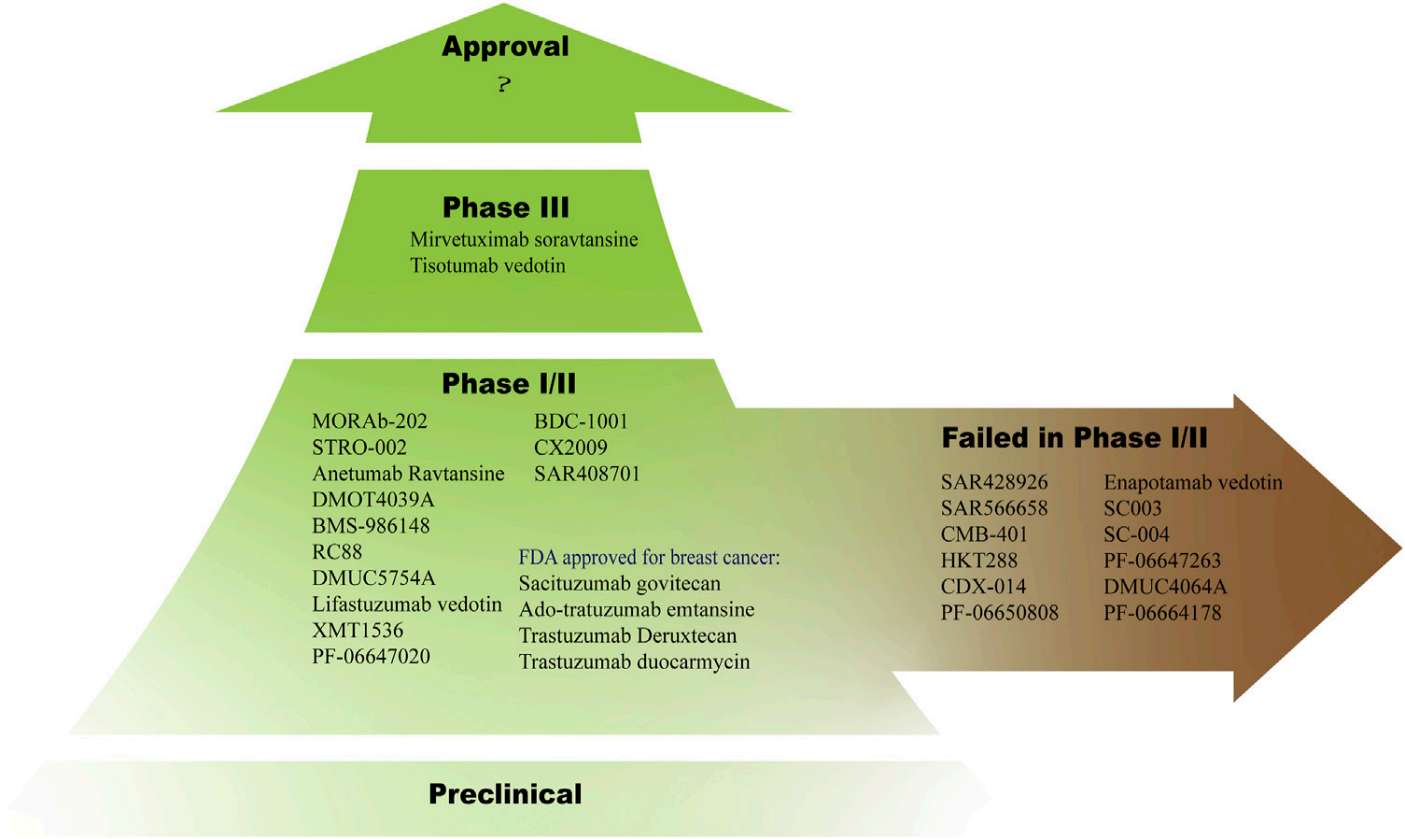
Dozens of ADCs are in clinical trials, most of them are in phase I or II and some of them have failed in this phase.

In gynecological oncology, new targets are continuously identified, prompting the design of novel treatments. After clinical trials, however, several ADCs in Table 4 have been discontinued due to a lack of efficacy at tolerable doses, issues with safety, or other reasons (Figure 2).

**TABLE 4:** Discontinued ADCs in gynecologic oncology.

ADC	Targets	Antibodies	Linkers	Payloads	DAR	Clinical trial phase	Gynecologic cancer mainly face to
SAR428926	LAMP1	Humanized IgG1	Sulfo-PDB linker	DM4	/	I, treatment emergent adverse events	Ovarian cancer
SAR566658	CA6	Human IgG1	Sulfo-PDB linker	DM4	/	I/II, unknown	Ovarian and cervical cancer
CMB-401	MUC1	Engineered IgG4	Hydrazone linker	Calicheamicin	/	II, did not meet efficacy endpoint	Ovarian cancer
HKT288	Cadherin-6	Human IgG1	Sulfo-PDB linker	DM4	/	I, neurotoxicity	Ovarian cancer
CDX-014	TIM-1	Human IgG1	Protease-labile mc-val-cit-PABC	MMAE	4.5	I, unknown	Ovarian clear cell carcinoma
PF-06650808	NOTCH3	Humanized IgG1	Protease-labile mc-val-cit-PABC	Aur0101	3.8	I, re-prioritization	Ovarian cancer
Enapotamab vedotin	AXL	Humanized IgG1-kappa	Protease-labile valine-citrulline linker	MMAE	/	I/II, insufficient activity	Ovarian, endometrial, and cervical cancer
SC003	DPEP3	Humanized IgG1-kappa	Protease-cleavable valine-alanine dipeptide linker	PBD	/	I, lacked safety profile and tumor activity to continue	Ovarian cancer
SC-004	CLDN6/9	Undisclosed	Undisclosed	PBD	/	I, low tolerability and lack of activity	Ovarian cancer
PF-06647263	EFNA-4	Humanized IgG1	Hydrazone cleavable linker	calicheamicin	4.6	I, limited response	Ovarian cancer
DMUC4064A	CA125	Humanized IgG1	Protease-labile peptide linker	MMAE	2	I, unrelated to safety	Ovarian cancer
PF-06664178	TROP2	Humanized IgG1	Protease-labile valine-citrulline linker	Aur0101	2	I, toxicity	Ovarian cancer

### 3.9 ADCs in combination with immune checkpoint therapy

#### 3.9.1 ICI treatments require a T cell-inflamed tumor microenvironment

ICIs induce long-lasting responses and even cure in some patient. These drugs enhance existing anti-tumor immune responses by preventing tumors from inhibiting T cell activation and anti-tumor immunological memory. ICIs have shown some success against endometrial cancer and an ORR of 53.0%–57.1% was observed among patients with MSI-H/dMMR (Marabelle et al., 2020). PD-1 inhibitors are recommended for those with recurrent/metastatic PD-L1-positive cervical cancer. However, ICIs are not effective against ovarian cancer. T cell-specific inflammation of the tumor microenvironment may explain why melanoma and lung cancer demonstrate high response rates to ICIs while most other cancer patients respond poorly. “T cell-inflamed” (hot) tumors that already have an ongoing anti-tumor T-cell response are more responsive to ICIs while “immune excluded” (cold) tumors are less responsive (Maleki Vareki, 2018). Thus, ICI in combination with other drugs may represent a direction for the development of future tumor immunotherapies. ADCs can theoretically turn tumors from immunologically cold to hot, providing the T cell-inflamed microenvironment needed for ICI treatment (Torres and Emens, 2022). In the WSG-ADAPT trial, T-DM1 increased the number and density of tumor-infiltrating T cells in breast cancer patients (Müller et al., 2015).

#### 3.9.2 The mechanism and preclinical model of ICI-ADC combined treatment

One of the mechanisms by which cytotoxic compounds, such as metantrine and dorastatin, induce antitumor immunity is by directly activating and inducing the maturation of dendritic cells (DCs) and the production of proinflammatory cytokines (Martin et al., 2014; Müller et al., 2014). There is also evidence that topoisomerase I inhibitors can act as immunomodulators to activate DCs (Gavrilescu et al., 2020).

**FIGURE 3:**
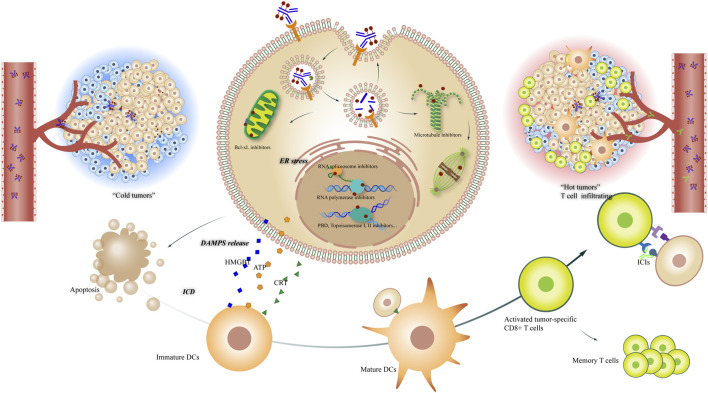
Potential mechanisms of ADCs in combination with tumor immunology therapy. Left: “cold tumor” without T cell infiltrating; right: “hot tumor” with active tumor-specific CD8^+^ T cells, offering a tumor microenvironment to ICIs; center: The different mechanisms of ADCs killing tumor cells, including Bcl-xL inhibiting, RNA spliceosome inhibiting, RNA polymerase inhibiting, DNA damaging and microtubule inhibiting, etc; down: The process that ICDs induce DCs maturing and T cell activating, leading to the convert of tumor microenvironment.

Cytotoxic compounds also induce antitumor immunity by promoting immunogenic cell death (ICD), a functionally unique response pattern that occurs after tumor cells have been treated with certain chemotherapeutic drugs, oncolytic viruses, physicochemical therapies, photodynamic therapy, and radiotherapy. The death of tumor cells promotes the exposure, active secretion, and passive release of various signaling molecules. These include damage-associated molecular patterns (DAMPs), such as calreticulin, high mobility group protein 1 (HMGB1), ATP molecules, and heat shock proteins (HSP70, HSP90). DAMPs released during ICD are recognized by pattern recognition receptors (PRRs) on DCs, causing activation, homing, and/or maturation, that results in the cross-presentation of tumor antigens to CD8^+^ cytotoxic T lymphocytes (CTLs) and ultimately activates both the innate and adaptive immune responses (Fucikova et al., 2020). Only a few chemotherapeutics, including anthracyclines and oxaliplatin, are ICD-inducing and are thus of particular interest for ICI combination treatments (Figure 3).

Some ADC payloads, such as maytansinoids and auristatins, and DNA alkylating agents such as pyrrolobenzodiazepines, can stimulate immune cells and enhance the anti-tumor efficacy of ICIs in preclinical models (Rios-Doria et al., 2017), which are shown to have a synergistic rather than an additive effect. For gynecologic cancer, mirvetuximab soravtansine induces ICD *in vivo*, as shown by the upregulation of ICD markers. Combined mirvetuximab soravtansine and pembrolizumab treatment have a synergistic antitumor effect that is dependent on CD8^+^ cells in a murine EOC model. These findings support the development of clinical trials to test the use of this drug combination in humans (Gavrilescu et al., 2020). Tumor cells treated with tisotumab vedotin are shown to release DAMPs and co-culture with allogeneic human PBMCs leads to innate immune cell activation and T cell proliferation *in vitro*. The combined use of tisotumab vedotin and pembrolizumab further enhances T cell proliferation and cytokine production. *In vivo* studies have shown that tisotumab vedotin treatment recruits F4/80+ and CD11^+^ innate immune cells to xenograft tumors. These data provide evidence for the immunomodulatory effects of tisotumab vedotin (Gray et al., 2020).

#### 3.9.3 Clinical data

Table 5 summarizes ongoing clinical trials which are evaluating the combined use of ADCs and ICIs for the treatment of gynecological tumors. One study assessed 14 patients with platinum-resistant ovarian cancer who were treated with both the PD-1 inhibitor, pembrolizumab (Keytruda), and mirvetuximab soravtansine (NCT02606305). The safety profile was consistent with the known profiles of each agent. The confirmed ORR was 43% (6/14 patients) and the overall median PFS of the trial was 5.2 months, with a median DOR of 30.1 weeks. The group with high FRα expression had a PFS of 8.6 months with a median DOR of 36.1 weeks. In contrast, the ORR of pembrolizumab monotherapy in PD-L1-positive ovarian cancer was only 11.5%, with a median PFS of 1.9 months (Matulonis et al., 2018). Combined use of tisotumab vedotin and pembrolizumab was tested among 33 patients with cervical cancer. The observed safety was generally consistent with a single agent. The confirmed ORR among 32 evaluable patients was 41%, with three (9%) CRs and ten (31%) PRs. The median time to response was 1.4 months and the median PFS was 5.3 months (Lorusso et al., 2022). A study assessing the combined use of trastuzumab emtansine and atezolizumab among patients with recurrent or persistent endometrial cancer is ongoing.

**TABLE 5:** ADCs in gynecologic oncology combined to immune checkpoint inhibitors in clinical trials.

ADCs	Immune checkpoint inhibitors	Conditions	NCT numbers
Mirvetuximab soravtansine	Pembrolizumab	Ovarian cancer	NCT02606305
	Pembrolizumab	Endometrial cancer	NCT03835819
Anetumab Ravtansine	Nivolumab, nivolumab, ipilimumab	Pancreatic cancer	NCT03816358
	Pembrolizumab	Mesothelioma	NCT03126630
BMS-986148	Nivolumab	Mesothelioma	NCT02341625
Tisotumab Vedotin	Pembrolizumab	Solid tumors	NCT03485209
	Bevacizumab, pembrolizumab	Cervical cancer	NCT03786081
Trastuzumab emtansine	Pembrolizumab	Breast cancer	NCT03032107
	Atezolizumab	Breast cancer	NCT02924883, NCT02605915
	Atezolizumab	Endometrial cancer	NCT04486352
Trastuzumab deruxtecan	Pembrolizumab	Breast cancer, non-small cell lung cancer	NCT04042701
	Nivolumab	Breast cancer, urothelial carcinoma	NCT03523572
	Durvalumab	Non-small cell lung cancer	NCT04686305
	Durvalumab	Breast cancer	NCT04556773, NCT04538742
BDC-1001	Nivolumab	HER2 positive solid tumors	NCT04278144
Sacituzumab Govitecan	Pembrolizumab	Urothelial cancer	NCT03547973
	Pembrolizumab	Breast cancer	NCT04468061, NCT04230109, NCT04448886, NCT05382286
	Pembrolizumab	Non-small cell lung cancer	NCT05186974
	Atezolizumab	Breast cancer	NCT04434040, NCT03424005
	Atezolizumab	Urothelial carcinoma, bladder cancer	NCT03869190
	Ipilimumab, nivolumab	Urothelial carcinoma	NCT04863885
	Avelumab	Breast cancer	NCT03971409
	Avelumab	Urothelial carcinoma	NCT05327530

## 4 Future directions and conclusion

Restrictive off-target toxicity is the main safety issue currently faced by ADCs, with fatigue, nausea, diarrhea, anorexia, vomiting, and peripheral sensory neuropathy being the primary AEs. Notably, ocular toxicity and pulmonary damage appear to be ADC-specific. In resistance, ADCs have resistance mechanisms that are like those of their individual components. Endocytosis, lysosomal function, and medication efflux pumps are also linked to resistance. The development of novel cytotoxic agents and the innovation of linkers and conjugation technology will help to break through the competition of ADCs.

Identifying additional agents that can generate “immune-synergy” has been a major focus since the initial successes of ICIs. Some ADCs payloads can induce ICDs. However, lymphopenia and neutropenia, known adverse effects of microtubule-targeting agents, are a concern of combined ADC and ICI treatment, which can influence anti-tumor immunity. Clinical trials are required to show whether combination therapies can benefit patient populations that do not respond well to monotherapy.
